# Faculty Perceptions on the Roles of Mentoring, Advising, and Coaching in an Anesthesiology Residency Program: Mixed Methods Study

**DOI:** 10.2196/60255

**Published:** 2025-01-21

**Authors:** Sydney Nykiel-Bailey, Kathryn Burrows, Bianca E Szafarowicz, Rachel Moquin

**Affiliations:** 1 Department of Anesthesiology, Washington University School of Medicine, 660 S Euclid Avenue, Saint Louis, MO, United States, 1 3149565620; 2 National Coalition of Independent Scholars, Independent Scholar 432 Division, Oregan City, OR, United States

**Keywords:** coaching, faculty perceptions, mentoring, perception, medical education, anesthesia, modality, support, Washington University, university, coaching skills, training, culture change, culture, flexibility, systematic framework

## Abstract

**Background:**

Mentoring, advising, and coaching are essential components of resident education and professional development. Despite their importance, there is limited literature exploring how anesthesiology faculty perceive these practices and their role in supporting residents.

**Objective:**

This study aims to investigate anesthesiology faculty perspectives on the significance, implantation strategies, and challenges associated with mentorship, advising, and coaching in resident education.

**Methods:**

A comprehensive survey was administrated to 93 anesthesiology faculty members at Washington University School of Medicine. The survey incorporated quantitative Likert-scale questions and qualitative short-answer responses to assess faculty perceptions of the value, preferred formats, essential skills, and capacity for fulfilling multiple roles in these support practices. Additional areas of focus included the impact of staffing shortages, training requirements, and the potential of these practices to enhance faculty recruitment and retention.

**Results:**

The response rate was 44% (n=41). Mentoring was identified as the most important aspect, with 88% (n=36) of faculty respondents indicating its significance, followed by coaching, which was highlighted by 78% (n=32) of respondents. The majority felt 1 faculty member can effectively hold multiple roles for a given trainee. The respondents desired additional training for roles and found roles to be rewarding. All roles were seen as facilitating recruitment and retention. Barriers included faculty burnout; confusion between roles; time constraints; and desire for specialized training, especially in coaching skills.

**Conclusions:**

Implementing structured mentoring, advising, and coaching can profoundly impact resident education but requires role clarity, protected time, culture change, leadership buy-in, and faculty development. Targeted training and operational investments could enable programs to actualize immense benefits from high-quality resident support modalities. Respondents emphasized that resident needs evolve over time, necessitating flexibility in appropriate faculty guidance. While coaching demands unique skills, advising hinges on expertise and mentoring depends on relationship-building. Systematic frameworks of coaching, mentoring, and advising programs could unlock immense potential. However, realizing this vision demands surmounting barriers such as burnout, productivity pressures, confusion about logistics, and culture change. Ultimately, prioritizing resident support through high-quality personalized guidance can recenter graduate medical education.

## Introduction

The current landscape of medical education is influenced by both medical culture and shifting demographics among learners. Factors such as medical provider burnout [1], a nationwide shortage of medical staff [2], and the evolving characteristics of different generations of learners are reshaping medical education [3]. It is imperative that the well-being and guidance of learners, both personally and professionally, are recentralized as the core of medical education. Emphasizing principles such as advising, mentoring, and coaching is crucial to support learners in their journey toward academic and personal fulfillment. These principles should be thoroughly examined and reevaluated to empower learners to pursue paths of academic and personal success, foster self-assessment, ensure a nurturing learning environment, and encourage a commitment to lifelong learning [1,2]. The objective of this paper is to examine the attitudes and experiences of clinical-academic anesthesiology faculty with respect to their understanding and practice of mentoring, advising, and coaching. Our aim is to identify key themes that more clearly define these roles within medical education, as well as to elucidate potential barriers to their implementation and sustainability. Furthermore, we seek to understand faculty perspectives on the need for formalized educational support in these areas. We anticipate that the insights gained from this study could be broadly applicable across the graduate medical education spectrum, particularly as the focus in education increasingly shifts toward professionalism and well-being.

The education and welfare of medical residents hinge upon a multifaceted network of connections. Residents at different stages of their training will necessitate varying forms of engagement: mentoring, advising, or coaching. While these 3 avenues are distinct, they all share the common aim of nurturing education, wellness, and career progression [2,3]. Each approach serves its unique purpose and uses diverse methodologies [2]. Identifying the most suitable modality for the learner is paramount. Facilitators must adeptly involve themselves and customize sessions to ensure that expectations and objectives resonate with the learner [2].

Traditionally, mentoring has been the primary means of providing guidance [4]. It entails a sustained personal relationship between mentor and mentee, with the learner’s overarching aspirations guiding the interaction. Conversations, career mapping, and counsel are derived from the mentor’s experiences and expertise [2,3]. Typically, mentors possess knowledge in the pertinent field and share their insights with the learner. The mentor guides sessions, posing direct questions with long-term goals as the focal point. In residency education, mentoring often follows a structured format, though informal mentorships may naturally evolve. Institutions may request mentors to provide feedback or document these sessions for accreditation purposes [2,3].

Advising typically comprises a single, informal session focused on a specific issue or inquiry. The advisor leads the session and provides solutions or strategies based on their own experiences. The learner has the autonomy to decide whether to heed the advice. Unlike mentoring, a sustained relationship is not necessarily a prerequisite for advising, and subsequent follow-up is usually with independence and self-driven by the needs of the advisee [5]. Advisors may possess limited insight into the learner’s personal or academic strengths and weaknesses, resulting in advice limited to specific scenarios [6].

Academic coaching differs from advising and mentoring in that it prioritizes the learner’s agency. Coaches refrain from offering advice or engaging in decision-making. Instead, their role is to facilitate self-discovery and create a supportive atmosphere for self-assessment and future planning [2]. Coaches assist learners in identifying actions that may lead to success or failure. Unlike mentors and advisors, coaches may not necessarily possess expertise in the medical field. Coach engagement is supported by actively listening to the learner and offering questions to encourage self-awareness. Coaching fosters a consistent, enduring relationship characterized by an educational partnership between coach and learner [2].

No single form of guidance is adequate to meet the needs of today’s students, and students’ needs evolve as they move through residency [7]. Faculty must be facile in their ability to intuit what type of guidance is appropriate for a specific student or situation, and be able to provide that guidance or refer the student to someone who can [8]. For this reason, faculty development programs play a crucial role in supporting faculty as they rise to meet the challenges of guiding trainees, and faculty training in these support modalities may be lacking [9]. Training educators on how to target student needs by using the most effective guidance strategy will help decrease role confusion [8]. Training and developing faculty in advising, mentoring, and coaching help cultivate an ongoing culture of scholarship [10] and can help faculty navigate the competing challenges of their clinical and nonclinical roles [11]. Faculty report that lack of support from leadership and lack of proper training are barriers to their role as advisors, coaches, and mentors [11], and training and assessment tools for faculty members are crucial [7,9].

## Methods

### Study Design

A survey (Multimedia Appendix 1) was sent to 93 Washington University School of Medicine Anesthesiology clinical educator faculties. This target population was used as a convenience sample, representing a cohesive cohort with consistent interactions with trainees. This survey was developed based on core competencies and conceptual differentiations outlined for the roles of advisors, coaches, and mentors in medical education [5,6,8,9]. Drawing from Wolff et al [9], support modality definitions and key characteristics were designed to reflect critical distinctions regarding focus, relationship context, longevity, skill sets, and objective alignment [9]. Survey questions were formulated to assess physician perspectives across these theoretical domains for each resident support role.

A group of coaching experts within the Department of Anesthesiology was selected to create a novel survey tool. To facilitate the design and construction of the survey instrument, the research team used a modified Delphi technique, a widely recognized method for achieving consensus among experts. A subset of academic faculty was invited to participate in a pilot study aimed at testing multiple dimensions of the survey’s implementation. This pilot study served several purposes: (1) to ensure the clarity and comprehensibility of the survey questions, (2) to evaluate the technical functionality of the survey platform, and (3) to assess the feasibility of applying inductive thematic analysis to the pilot data. Through iterative revisions and rounds of expert feedback, the survey underwent several modifications to enhance both face validity and content validity. The final version of the survey, which reflects the culmination of this rigorous development process, is presented in Multimedia Appendix 1. The survey is composed of 2 Likert 5-point scale quantitative items and 11 qualitative open-ended questions.

Quantitative items examined perceptions of importance and optimal configurations applying the principles of Wolff et al [9] regarding situational demands and need for role clarity [9]. Quantitative data were collected using the REDCap (Research Electronic Data Capture) Consortium platform (Vanderbilt University), a secure web-based application designed to support data capture for research studies. Faculty received the voluntary survey through department email, no incentives offered, and faculty log-in prevented duplicate entries. Data were analyzed using descriptive statistics. Qualitative questions elicited feedback on specialized skills, training interests, and implementation barriers grounded in advising, coaching, and mentoring competency frameworks [5-9]. The sequence of survey topics reflects established theory comparing and contrasting these support avenues [6-8]. An inductive qualitative analysis was conducted, using the Braun and Clarke [12] 6-phase approach to thematic analysis. This methodological framework, widely used in qualitative research, ensures both the flexibility and rigor required for the interpretative analysis of complex datasets. The 6 stages—familiarization with data, generating initial codes, searching for themes, reviewing themes, defining and naming themes, and writing up—provide a structured yet adaptable framework for data interpretation [12]. Qualitative data were collected through open-response questions included in the REDCap survey. The text from these open-response questions was analyzed using the Dedoose coding themes platform.

The process begins with open coding to identify initial patterns within the data. Codes were then further examined to uncover relationships, allowing for the grouping of related codes into broader thematic categories. Subsequently, these groups were analyzed to identify overarching themes that reflect deeper insights into the data. This iterative process was designed to ensure a comprehensive exploration of the qualitative data and enhance the interpretive depth of the analysis.

To ensure the reliability of the findings, at least two independent researchers reviewed and coded all data. The initial coding and preliminary analysis of the qualitative data was conducted by 1 author (SN-B), using Dedoose—a cloud-based software platform designed to facilitate mixed methods analysis. After the initial coding phase, 2 members of the research team engaged in collaborative discussions to reconcile coding discrepancies and synthesize their interpretations. This process of researcher triangulation not only strengthens the credibility of the findings but also helps to ensure that the emerging themes are robust and reflect the nuances present in the data [13].

The survey contained a brief textual description of the difference between the roles of mentor, advisor, and coach, respondents. Respondents were asked how important they thought each role is in graduate medical training, whether 1 individual can fulfill all 3 roles, what kind of training is needed for faculty to perform these roles, and whether resident needs for different forms of faculty relationship change over time. In addition, faculty were asked if they had ever performed any of the 3 roles. Questions were both quantitative (responses on a 5-point Likert scale) and qualitative (open-ended short responses). A total of 41 surveys were completed (44% response rate).

### Ethical Considerations

This study used both quantitative and qualitative data collection and analysis. This study was approved by the Institutional Review Board at Washington University (202310164). Faculty were informed about this study via an initial email announcement, followed by 2 reminder emails. Informed consent was obtained with faculty selecting “accept” on the survey; the ability to opt out was provided. Electronic data were password protected, encrypted, and transmitted using recognized security for electronic submission. No compensation was provided.

## Results

### Roles

Respondents had varying opinions about the importance of mentoring, advising, and coaching in graduate medical education. Mentoring was seen as most important, with 88% of respondents indicating that they agreed or strongly agreed that it was important, and coaching was seen as less important, with only 78% of respondents indicating agreement or strong agreement that it was important (Table 1).

**Table 1.** Importance of mentoring, advising, and coaching.

	Agree or strongly agree, n (%)
Coaching	32 (78)
Advising	34 (83)
Mentoring	36 (88)

In total, 90% of respondents agreed that 1 faculty member could fulfill two or more roles for a single resident. For example, respondent 2 explained, “Faculty can possess more than one skill set and/or the relationship between a faculty and resident may benefit from a multi-faceted focus once trust has been developed.” However, others noted that there may be conflicts between roles and that the unique skills required for each role are not always possessed by the same person. Respondent 3 noted, “This works sometimes, I think, but can’t dependably work all the time. Some faculty are better at one role or another. Obviously, some coaching and advising can only be done by faculty with certain skills or areas of expertise.”

Additionally, respondents noted the role faculty mentoring and coaching play in recruitment and retention efforts for faculty and trainees. For example, respondent 3 noted, “if it were made clear that we offered thoughtful assignment of each of these roles, with examples for coaching and advising, I think that would likely be seen as a significant benefit.” Others agreed that providing these roles to residents in a systematic way would be beneficial for recruitment, but noted barriers to implementation, as respondent 33 explained, “I think that these three roles are important to recruit residents for fellowships and faculty. Fostering a supportive environment through these roles is very important for recruitment; however, other factors such as the job market and hours worked often overshadow these aspects in recruiting.”

### Training

Most respondents agreed that specialized training in all 3 roles was important, especially for coaching, which was seen as requiring a unique skill set. Formal training for all 3 roles was endorsed, especially for coaching. Respondents noted that the skills required for the roles came naturally to some faculty. For the advising role, having career experience and expertise in the graduate education process was seen as especially useful. For example, respondent 10 noted, “Knowing the residency experience well and knowing what challenges residents face. Additionally, it’s important to know career options after.” Mentoring was regarded as being based on relationship building and interpersonal skills, as well as necessitating emotional intelligence. Respondents reflected that mentorship involves skill sets not necessarily embedded in clinical training. Respondent 21 explained, “Teach the teacher/instructor courses are helpful. Being a good clinician and/or researcher do not provide us the skills of being a good teacher. A bit of more understanding, empathy, and psychological support are necessary for knowing ourselves better and using these abilities for others. Patience, more listening, time, sharing experiences, sometimes coming up with a challenging scenario to discuss, widen the horizon, show other possibilities never thought of before as options.”

Respondents indicated that they would be interested in targeted training. Coaching (63%) was the highest, however, respondents were less interested in specialized training in advising and mentoring skills (Table 2).

**Table 2.** Interest in specialized training in coaching, advising, and mentoring.

	Interest in specialized training, n (%)
Coaching	29 (63)
Advising	17 (41)
Mentoring	16 (39)

### Experience

Nearly 88% of respondents had fulfilled one or more of these roles in their career, and they noted that holding all 3 roles was personally and professionally rewarding. Of the 36 faculty members who reported fulfilling these roles in the past, 15 (42%) mentioned the satisfaction of watching students progress through their training and career. Coaching was noted as being the most challenging, but also the most rewarding. For example, respondent 22 said, “Honestly, I think that serving in this role for strong residents is one of the most rewarding parts of my job. I love to see people be successful in their careers.”

### Barriers

Respondents identified barriers to faculty engaging in quality mentoring, coaching, and advising, which included faculty burnout, time limitations, and confusion about roles, responsibilities, and expectations. Respondent 10 said, “The residents have so many rotations. It’s rare to have consistent clinic time to coach and mentor/advise. Coaching off hours is very time consuming.” Lack of time was mentioned by 68% of respondents, for example as respondent 29 explained “I was a terrible mentor. Never could find time to meet with my mentee.”

Respondents had mixed responses about whether the national anesthesia provider shortage had impacted their engagement with or performance of any of these roles. Respondents noted lack of time in general, and lack of protected time more specifically, as factors influencing their ability to engage in these roles, and some attributed the challenge with time to provider shortage. For example, respondent 17 said, “The shortage has decreased faculty time to provide these aspects, may be important for departments to assign a subgroup of faculty to serve these roles so time is protected.”

Table 3 presents the results of the thematic analysis, offering a detailed synthesis of the emergent themes and subthemes derived from the qualitative data. The richness of respondent narratives facilitated a comprehensive exploration, allowing for nuanced insights into the key thematic categories. These findings provide a robust framework for understanding the underlying patterns and relationships within the data, supporting the depth and validity of the analysis.

**Table 3.** Main themes and representative quotes.

Theme and subtheme		Representative quotes
**Roles**		
	Faculty can perform multiple roles	“Faculty can possess more than one skill set and/or the relationship between a faculty and resident may benefit from a multi-faceted focus once trust has been developed.” [Respondent 2] “Different skill sets are needed and faculty may possess one or many of the skill sets needed.” [Respondent 9] “I believe the necessary skills can be learned and employed by a single person. It also depends upon the mentee’s needs and the qualities of their relationship with the mentor/advisor/coach.” [Respondent 5] “A faculty member can take different roles throughout the 4 years that a trainee is counseled. I find that interns need mentoring and advising, as the resident progresses coaching and mentoring is important.” [Respondent 16]
	Faculty cannot perform multiple roles	“This works sometimes, I think, but can’t dependably work all the time. Some faculty are better at one role or another. Obviously, some coaching and advising can only be done by faculty with certain skills or areas of expertise.” [Respondent 3] “Sometimes the line between just providing feedback for a specific case as an advisor can be hard if you are also a mentor to that person.” [Respondent 37] “Different goals and different time frames over which those goals are realized. The trainee asking advising may be frustrated by a “mentoring” approach. Some great career mentors may not have the specific sub-specialty background for focused advising.” [Respondent 7]
**Training**		
	Request for formal education or faculty development	“I think at least some sort of education on how to be an advisor would be helpful.” [Respondent 1] “Teach the teacher/instructor courses are helpful. Being a good clinician and/or researcher do not provide us the skills of being a good teacher. A bit of more understanding, empathy, and psychological support are necessary for knowing ourselves better and using these abilities for others. Patience, more listening, time, sharing experiences, sometimes coming up with a challenging scenario to discuss, widen the horizon, show other possibilities never thought of before as options.” [Respondent 21] “Coaching should require some training/knowledge of professional coaching, which is more structured that mentorship or career advising which can be more informal.” [Respondent 4] “Didactics/workshops/peer mentoring needed.” [Respondent 31] “Training focused to the knowledge and skillset as well as teaching techniques and current best practices.” [Respondent 2] “Structured professional coaching training.” [Respondent 6]
**Experiences**		
	Mentor role	“I was a terrible mentor. Never could find time to meet with my mentee.” [Respondent 29] “Mentoring has been the most rewarding, coaching second. Advising feels limited and one-directional.” [Respondent 5]
	Coach role	“All three - coaching seems to be the most challenging.” [Respondent 7] “I have played all 3 roles during my time as an educator. The coaching roles are always the most rewarding. The ability to guide residents through self-discovery is extremely rewarding. I find that coaching residents later in their training prepares them for being faculty and having a successful trajectory.” [Respondent 17] “I have been a coach and an advisor. Coaching is extremely rewarding.” [Respondent 39] “Primarily coaching, which I found rewarding when a trainee felt our interaction was beneficial through skill-based or confidence improvements.” [Respondent 41]
	Advisor role	“Advising in clear goal-directed tasks, such as a conference, abstract, paper.” [Respondent 8] “I have served as a mentor and advisor, both of which were very rewarding. I felt that it made it easier to discuss topics at work that we may otherwise would not have brought up. I also felt satisfaction getting to know the trainees better and become more a part of their lives.” [Respondent 27] “Clinical teaching while supervising trainees fulfills the “advisor” role. I was also a designated faculty mentor for a clinical fellow.” [Respondent 34] “Clinical teaching while supervising trainees fulfills the advisor role.” [Respondent 33]
	Combination of roles	“Have provided all three of these roles in different capacities. I enjoy fostering learning with the goal of being the attending I wish I had as a trainee.” [Respondent 33] “Yes, I feel that I serve as an advisor to residents and mentor to fellows.” [Respondent 20] “I would say informally on day-to-day basis interactions with residents and fellows, yes for all 3. Advisor more than mentor more than coach. It is rewarding when it seems welcomed and appreciated by the residents and fellows and I can see them grow and improve. It is frustrating when I am putting in the effort/trying to do these things and the trainees are not receptive, not appreciative, or feel as though I am being too particular or micromanaging.” [Respondent 35] “I have provided all 3. The coaching roles are always the most rewarding. The ability to guide residents through discovery is extremely rewarding.” [Respondent 20]
	Recruitment role	“The biggest drivers right now for recruitment are time and money. The biggest long-term satisfaction will come from deeper meaning. Using the relationships in these roles may help highlight some of these deeper meanings and may help recruit fellows and faculty if they have the sense that this is best for themselves and their families. At the same time, there has to be felt and sustained room for the individual to act on these deeper meaningful insights. Solving for individual growth requires commitment from the system as well as the individual.” [Respondent 9] “Yes. When residents can see faculty care about their education and also enjoy working here it’s easier to recruit. “ [Respondent 20] “Mentorship and coaching require a relationship, that may be beneficial for recruitment.” [Respondent 17] “A structured mentor/coaching program would be very appealing to most applicants.” [Respondent 31]
**Barriers**		
	Discrete roles	“If role/project is not clearly defined, could cause some confusion. Time.” [Respondent 1] “Mentorship is often a friendly and personal relationship, which could make it harder to, for example, challenge the mentee in a coaching scenario. Very specific example - perhaps a mentee would feel uncomfortable doing mock oral boards with their mentor, if they’re relatively advanced in training, but early in the oral boards prep process.” [Respondent 3] “Different goals and different time frames over which those goals are realized. The trainee asking for advising may be frustrated by a “mentoring” approach. Some great career mentors may not have the specific sub-specialty background for focused advising.” [Respondent 7]
	Time	“Time and lack in continuous interactions with the resident.” [Respondent 18] “Time to meet with the trainee and to establish a relationship.” [Respondent 14] “Time and managing the balance btw one’s professional responsibilities and taking on additional responsibilities that the above would entail.” [Respondent 6] “The residents have so many rotations. It’s rare to have consistent clinic time to coach and mentor/advise. Coaching off hours is very time-consuming.” [Respondent 10]
	Burnout	“It would be a good recruitment tool but difficult to deliver in near future with current staffing shortages and burn-out among faculty members. In practice, it would require significant training, time, and effort to optimize and ensure an equal experience among trainees. Remuneration could increase participation but doesn’t get around the issue of lack of time.” [Respondent 12] “Yes, particularly for faculty. Relatively little resources currently to develop faculty. More investment needed to reduce the chance of burnout/disengagement/attrition to other practices.” [Respondent 31] “We are all strapped for time and burnt out.” [Respondent 40]
	Anesthesia shortage	“These 3 are probably even more important for our trainees and may be beneficial to expand these past trainees and onto faculty as well. The shortage has decreased faculty time to provide these aspects, may be important for departments to assign a subgroup of faculty to serve these roles so time is protected.” [Respondent 17] “I think that with the shortages, faculty have taken on more solo assignments and have overall less contact with the residents and don’t get to know them as well.” [Respondent 36]

## Discussion

### Overview

This study explored perceptions of anesthesia faculty regarding the roles of mentoring, advising, and coaching in graduate medical education. The results highlight the perceived benefits of these practices as well as barriers to implementation. Anesthesia residency is unique in its internship, and a vast majority of education and interactions with faculty occurs at bedside in the operating room. Medical training and trainee progression differ across disciplines. This study focuses specifically on anesthesia faculty and a single institution, which overall limits generalizability.

### Principal Findings

The survey results indicate that faculty view mentoring, advising, and coaching as important for resident education and development. These practices have been shown to improve resident well-being, promote career planning, facilitate reflection and self-assessment, and identify knowledge gaps [5,6]. Furthermore, implementing structured programs in these areas can aid recruitment and retention of both residents and faculty.

Of the 3 roles faculty partake in, there is a consensus on the importance of mentoring throughout training and prioritizing this role over advising and coaching. However, the data suggests a significant interest in specialized training for coaching versus roles in advising and mentoring. Investigating the differences in practice versus desire, recurrent themes of time and experience were identified. Although the roles as a mentor, advisor, and coach can overlap, a majority of the cohort indicated they prioritize mentoring given the noted constraints of time and experience.

### Implications of Findings

To actualize these practices, each department must clearly define the roles of mentor, advisor, and coach. Expectations, training requirements, and time commitments should be delineated. Assignments of roles can be made between faculty and residents based on alignment of career goals, personalities, and logistics. Protected nonclinical time should be designated for these meetings separate from clinical work. Success stories and positive impacts on residents should be tracked and celebrated.

### Comparison to the Literature

Recurrent themes were identified when comparing to other literature, such as the establishment of a clear definition and terms of each role. This would help faculty facilitate their approach to the learner needs [5,8]. Additional repeated themes of the overlap in roles, limitations in time, and experiences were highlighted in other studies in reference to mentorship, advising, and coaching [5,10]. Anesthesiology training presents challenges specific to the discipline, which can be generalized to medical training programs at other institutions. There has been an increased productivity within the academic institutions leading to less bedside education opportunities and difficulty establishing dedicated time for routine meetings with trainees.

### Limitations

This study has several limitations. First, this study was based on a single survey with a 44% response rate, which may limit the generalizability of the findings. Nonresponders may have had different perspectives on the importance and implementation of mentoring, advising, and coaching. Second, this study was conducted at a single academic medical center, so the results may not be representative of other institutions. Additionally, this study was solely conducted with anesthesia faculty. Other specialties may not portray the same obstacles and constraints in fulfilling the roles of mentorship, advising, and coaching. The learning environment and progression through training also differ between anesthesiology and other specialties, which limits the generalizability across disciplines. The limited time and consistency with faculty may lead to less specific demands from trainees and unfulfillment from educators. Third, the survey relied on self-reported perceptions and experiences, which are subject to recall bias and social desirability bias. Fourth, this study did not explore the perspectives of residents themselves on these support modalities. Future research should examine resident experiences with and preferences for mentoring, advising, and coaching. Finally, while this study identified perceived barriers to implementing these practices, it did not evaluate specific strategies for overcoming these obstacles. Further work is needed to develop and test interventions to enhance faculty engagement in resident support roles.

### Conclusion

Addressing barriers such as faculty burnout, role ambiguity, time constraints, and the need for specialized training is critical for the success of mentoring, advising, and coaching initiatives. Implementing comprehensive faculty development programs aimed at enhancing skills in these domains is essential, particularly for coaching, which requires distinct pedagogical approaches. The recruitment and retention of faculty, as well as their career longevity, may be positively influenced by the intrinsically rewarding nature of relationships with trainees.

To facilitate meaningful faculty engagement, institutional leadership must ensure protected time for participation in these activities without detriment to clinical productivity. Moreover, a cultural shift may be necessary in programs that place disproportionate emphasis on service obligations, potentially at the expense of educational and developmental support for residents. Prioritizing resident education and well-being can contribute to improved morale and overall program satisfaction.

By investing in faculty development, enhancing institutional infrastructure, and fostering a culture that values educational alliance, graduate medical education programs can realize significant benefits from high-quality mentoring, advising, and coaching relationships. Such investments are pivotal for advancing the professional development of both faculty and trainees, ultimately enhancing the overall quality of medical education.

## Supplementary material

10.2196/60255 Multimedia Appendix 1 Coaching, mentoring, and advising survey.

## Abbreviations

REDCap: Research Electronic Data Capture

## References

[1] Țăranu SM, Ștefăniu R, Rotaru TȘ (2023). Factors associated with burnout in medical staff: a look back at the role of the COVID-19 pandemic. Healthcare (Basel).

[2] GlobalData Plc (2024). The complexities of physician supply and demand: projections from 2019 to 2034.

[3] Plochocki JH (2019). Several ways generation Z may shape the medical school landscape. J Med Educ Curric Dev.

[4] Farkas AH, Allenbaugh J, Bonifacino E, Turner R, Corbelli JA (2019). Mentorship of US medical students: a systematic review. J Gen Intern Med.

[5] Marcdante K, Simpson D (2018). Choosing when to advise, coach, or mentor. J Grad Med Educ.

[6] Deiorio NM, Hammoud MM (2024). Coaching in medical education: accelerating change in medical education consortium.

[7] Alisic S, Boet S, Sutherland S, Bould MD (2016). A qualitative study exploring mentorship in anesthesiology: perspectives from both sides of the relationship. Can J Anesth/J Can Anesth.

[8] Santiesteban L, Young E, Tiarks GC (2022). Defining advising, coaching, and mentoring for student development in medical education. Cureus.

[9] Wolff M, Deiorio NM, Miller Juve A (2021). Beyond advising and mentoring: competencies for coaching in medical education. Med Teach.

[10] Reid MB, Misky GJ, Harrison RA, Sharpe B, Auerbach A, Glasheen JJ (2012). Mentorship, productivity, and promotion among academic hospitalists. J Gen Intern Med.

[11] Jha P, Quinn B, Durbin S, Bhandari S (2019). Perceptions of junior faculty in general internal medicine regarding mentoring medical students and residents in scholarly projects. J Gen Intern Med.

[12] Peel KL (2020). A beginner’s guide to applied educational research using thematic analysis. Pract Assess Res Eval.

[13] Patton M (2015). Qualitative Research and Evaluation Methods.

