# Supplementary material for: Faculty Perceptions on the Roles of Mentoring, Advising, and Coaching in an Anesthesiology Residency Program: Mixed Methods Study
Source: JMIR Med Educ. 2025 Jan 21;11:e60255. doi: 10.2196/60255 (PMC11774320; doi:10.2196/60255)
Supplement: Multimedia Appendix 1 [file mededu-v11-e60255-s001.docx]

Please complete the following survey exploring the roles of mentor, advisor, and coach as pertains to graduate medical education.

Top of Form

| Please reference the provided definitions as you respond to the survey.  Mentor: An experienced and knowledgeable individual who offers guidance and support. Key characteristics: 1. Builds a personal and trusting relationship with the mentee 2. Provides advice and wisdom 3. Offers encouragement, support, constructive feedback 4. Relationship is long-term 5. Shares networks and connections with the mentee  Advisor: Someone with specialized knowledge or expertise in a specific domain. Key characteristics: 1. Focused on addressing specific challenges or achieving specific objectives 2. Relationship is task-driven 3. May be ongoing or a one-time interaction  Coach: Helps an individual improve their performance and skills in a certain area. Coaches help engage and facilitate personal growth and self-discovery. 1. Employs active listening skills and powerful question techniques 2. Helps coachee set clear, measurable goals 3. Supports an environment of self-discovery 4. Supports the coachee in their development of actionable plans |
| --- |
| **Survey Questions**   1. Coaching by faculty is important to aid in the training of residents.     Strongly disagree  Disagree  Undecided  Agree  Strongly agree |
|  |
| 1. Mentoring by faculty is important to aid in the training of residents.     Strongly disagree  Disagree  Undecided  Agree  Strongly agree |
| 1. Can one faculty member fulfill 2 or more roles for the same resident: mentor, advisor, and coach?   Yes  No Explain |
| 1. What barriers are encountered that make it difficult to fulfill 2 or more roles? |
| 1. What specialized training is important for an individual to be an effective advisor in medical education? |
| 1. What specialized training is important for an individual to be an effective coach in medical education? |
| 1. What specialized training is important for an individual to be an effective mentor in medical education? |
| 1. Would you be interested in additional training in coaching?    Yes   No Explain |
| 1. Would you be interested in additional training in advising?     Yes  No Explain |
| 1. Would you be interested in additional training for mentoring?     Yes  No Explain |
| 1. Have you provided any of these 3 roles during your time as an educator? Which ones? In what ways did you find the experience rewarding? |
| 1. Has the national anesthesia provider shortage changed your approach to any of these roles? How? |
| 1. In your experience, do you think any of the 3 roles help or would help to recruit residents for Wash U fellowships or Wash U faculty? Explain why please. |

Bottom of Form

Top of Form
